# The Farrington Curve: A Commentary on How David Farrington Showed How to Prioritise the Most Harmful Offenders

**DOI:** 10.1002/cbm.2372

**Published:** 2025-01-17

**Authors:** Lawrence W. Sherman

**Affiliations:** ^1^ Institute of Criminology University of Cambridge Cambridge UK

It is unlikely that David Farrington ever saw what I name in this commentary as the ‘Farrington Curve’, which plots the combined seriousness and frequency of reported offending among the most harmful suspects in any population. It is also unlikely that he ever knew just how extreme the difference can be in cumulative harm between median offenders and the most extreme outliers. Even so, without Farrington's years of pondering and publishing on such issues, I doubt that Sir Mark Rowley, a Cambridge mathematics graduate and current Commissioner of Police of the Metropolis, would have even asked a vitally important question as he took office in 2022: *Who are the hundred most dangerous people in London*?

Fortunately, the work of David Farrington had already spread widely in British criminology and policing, at least among the discerning. That work, based on the 411 males from southeast London who David studied for much of his life (and theirs), revealed important differences among people who were either self‐reported criminals, convicted offenders or both. These differences went far beyond the orthodox UK Civil Service perspective on repeat offending as an either/or category, with nil regard to the difference between a bicycle theft and a murder or between one bike theft and two hundred.

As late as 2007, the UK Home Office told me that the only officially acceptable test of whether a justice innovation worked was the percentage of convicted offenders who were convicted a second time within a fixed time period (usually 2 years). Even while the Home Office economists had been developing a cost‐of‐crime weighting for common offences based on governmental expenditures per crime for each crime type (Brand and Price 2000), the policy officials would not accept a cost‐of‐crime reduction as a measure of reduced severity and frequency of crime. In response to my challenge, I was invited by the Home Office policy team to argue the point with a Home Office statistician, but the statistician agreed with me—and with David Farrington who had already written on the issue. Therefore, using the Home Office economists' estimates of cost‐of‐crime tariffs by offence category, the estimates by Shapland et al. (2008) were able to show that police‐led restorative justice conferences lowered repeat offending costs of crime substantially in three of our randomised controlled trials (L. W. Sherman et al. 2015).

The foundation for the Home Office economists' work had been laid decades ago by David Farrington. His 1987 *Crime and Justice* article showed how large the variance in the frequency of crime was across his 411 cases (Farrington 1987). That article also identified the need for criminology to create an index to show how *dangerous* the mix of any one person's offending was in relation to the relative seriousness of the variety of offence types. In a 1985 discussion of differences in seriousness of offending across individuals, David Farrington and I began to speculate about whether that dimension of criminality had even more extreme variance than frequency of crime measured as if ‘all crimes are created equal’. I pondered that discussion for years, prior to publishing a workable ‘Cambridge Crime Harm Index’ (CCHI) with Peter and Eleanor Neyroud (L. Sherman, Neyroud, and Neyroud 2016).

Once that index was in hand, so were the means of ‘stacking’ suspected offenders in rank order of the total seriousness of all crime alleged against them by victim reports to the police. Thus when Commissioner Rowley asked the question, the first Chief Scientific Officer at Scotland Yard (L. Sherman) was able to produce the metric Farrington had implied in 1987.

By plotting a universe of over 100,000 people over age 18 in 2022–2023 who had been named by victims and offenders as suspects of violence against women and girls (VAWG),^1^ our team at the Metropolitan Police found that only some 35,000 of named VAWG suspects had been accused of two or more separate crimes during the date range. Using that standard as a simple (if crude) means of screening out false accusations, the MPS team arranged in rank order all those two‐or‐more offence suspects based on the total CCHI scores for VAWG crimes of each suspect. The list of suspects was also limited to those who had a fingerprint‐verified identification number from the Police National Computer (PNC) system.

Using the Cambridge Crime Harm Index (L. Sherman, Neyroud, and Neyroud 2016) values of the days of imprisonment (for each VAWG offence category) as recommended by the national Sentencing Guidelines Council as the starting point for a sentencing decision, the MPS data analysts plotted this distribution of the ∼35,000 suspects by the sum of all CCHI scores for each crime reported by victims or witnesses (Figure 1 below, as presented in L. Sherman et al. 2024). This is what we should call the ‘Farrington Curve’, in honour of David Farrington's asking these questions of his 411 subjects—with “answers” from over 100,000.

**FIGURE 1:**
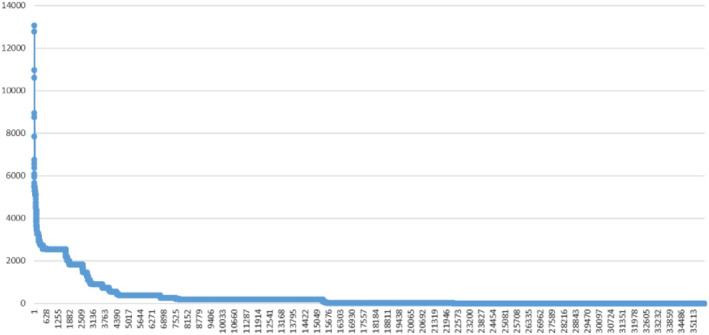
Distribution of VAWG harm by individual suspects.

## 1 Why Is the Farrington Curve Important?

When the VAWG curve we designed first appeared in my Scotland Yard email, I was astonished to see how extreme and steep it was. It seemed far more extreme than similar distributions of crime *counts* by places, even in the same population size range. When L. W. Sherman, Gartin and Buerger (1989), for example, plotted every one of the 115,000 street addresses in Minneapolis, they found the maximum number of predatory crime events reported to police by phone (911) to be 410. Yet when we consider London's VAWG crimes as varying in seriousness—and not just volume—what we find is a range of seriousness that stretches over 13,000 times higher than anyone named for one simple assault, with a sentencing starting point (CCHI Score) of 1 day imprisonment.

By comparison to a spatial metaphor in London's landscape, the highest building in the metropolis is the ‘Shard’, which towers 95 storeys above a landscape with many one‐storey buildings. Yet, the distance in a harm metric of days imprisonment shows 13,000 times more VAWG harm in the highest offenders than in the lowest or almost 137 times higher again relative to the 95‐to‐1 difference of the Shard from many buildings. The extreme distance between the highest harm offenders and those with only 1 day's prospective penalty is truly hard to comprehend.

Without appreciating this enormous extremity of difference between *very high* harm and the *highest* harming suspects, we cannot make sensible crime policy. We truncate ‘high’ risk levels into a single category that has a range of 1000 times higher at the top than at the bottom of ‘high’ harm, with a vast middle range of ‘high’ risk in between. Nevertheless, what the data show is that the peak of harm is so extreme that we are virtually ignoring it. We invest most resources in a little bit of public response for the vast number of reported crimes, including VAWG. What we do not even come close to doing is to invest in the *prevention* of high‐harm crimes with resources that are proportionate to the severity of harm as determined by the judges who impose the penalties.

## 2 How David Farrington Earned His Name on This Curve

David Farrington would be the first to say that science is incremental and that single‐person developments cannot be fairly named for only one of the developers. But Professor Farrington's legacy is a special case. He made so much out of the Cambridge Study in Delinquent Development (CSDD) that it shaped a generation of criminological thought. My thesis for this commentary is that until he pushed the CSDD onwards over decades of the life course of 411 people and their families, few public officials or criminologists even understood that there were major differences in harm and frequency of offences across offenders. And as the Farrington curve shows, the curve is especially skewed for highest total harm alleged.

Because we can all point to David's work as the first such analysis ever approaching this rank‐ordering in an offender population, we should put his name to the most visible outcome of that work (so far). His name will also remind us never, for example, to support any crude application of this analysis to sentencing guidelines, let alone as evidence of guilt in a trial of the facts. What he would do, however, is support the use of any harm index (or crime severity scores, as the Office of National Statistics calculates them) to *set priorities* for investing in crime prevention across victims, places and suspects. His later work on the costs of crime pointed in exactly that direction (Farrington and Welsh 2023).

Therefore, as we remember David and the life work he devoted to criminology, let us use the Farrington curve to help people see what David expected long ago that we would find together, eventually. All offenders are not alike. Their frequency and severity of offending vary enormously. So should our investments in preventing those crimes.

## Conflicts of Interest

The author declares no conflicts of interest.

## Data Availability

The author has nothing to report.

## Endnotes

^1^
On a list of offences agreed by the National Police Chiefs' Council (NPCC).
